# Sunitinib-induced morpho-functional changes and drug effectiveness in malignant solitary fibrous tumours

**DOI:** 10.18632/oncotarget.7523

**Published:** 2016-02-20

**Authors:** Rosalin D. Spagnuolo, Silvia Brich, Fabio Bozzi, Elena Conca, Chiara Castelli, Marcella Tazzari, Roberta Maestro, Monica Brenca, Ambra V. Gualeni, Annunziata Gloghini, Silvia Stacchiotti, Marco A. Pierotti, Silvana Pilotti, Tiziana Negri

**Affiliations:** ^1^ Laboratory of Experimental Molecular Pathology, Department of Diagnostic Pathology and Laboratory, Fondazione IRCCS Istituto Nazionale dei Tumori, Milan, Italy; ^2^ MOSE-DEA, University of Trieste, Trieste, Italy; ^3^ Department of Experimental Oncology and Molecular Medicine, Unit of Immunotherapy of Human Tumours, Fondazione IRCCS Istituto Nazionale dei Tumori, Milan, Italy; ^4^ Experimental Oncology 1, Centro di Riferimento Oncologico, Aviano, Italy; ^5^ Department of Diagnostic Pathology and Laboratory, Fondazione IRCCS Istituto Nazionale dei Tumori, Milan, Italy; ^6^ Adult Mesenchymal Tumour and Rare Cancer Medical Oncology Unit, Cancer Medicine Department, Fondazione IRCCS Istituto Nazionale dei Tumori, Milan, Italy; ^7^ Scientific Directorate, Fondazione Città della Speranza, Padua, Italy

**Keywords:** malignant solitary fibrous tumours, efficacy of sunitinib, autophagy, immune cells

## Abstract

Sunitinib improves the outcomes of patients with solitary fibrous tumours (SFTs). The aim of this study was to investigate and contextualise sunitinib-induced morpho-functional changes in order to gain insights into the drug's mechanism of action.

To this end, four surgical specimens obtained from two sunitinib-responsive patients with malignant SFT, and one primary cell culture obtained from fresh tumoral tissue and its stabilised cell line, were studied by means of immunohistochemistry, bright field *in situ* hybridisation, immunofluorescence/confocal microscopy, and biochemistry.

The post-sunitinib surgical samples were characterised by two biologically relevant morpho-functional changes: clear areas and necrotic foci. The first were associated with the attenuation/loss of PDGFRB expression and decreased mTOR signalling, and corresponded to a pathological response. The second were associated with the over-expression of PDGFRB and VEGFA, strong mTOR signalling activation, and the appearance of HIF1α expression, hallmarks of pathological progression. The analysis clearly showed that sunitinib reduces the vascular supply network and inhibits tumoral cells. It also either induces autophagy, thus favouring drug response, or impairs autophagy as a result of lysosome sequestration, thus favouring disease progression. These distinct autophagic events were associated with different myeloid immune contextures. Finally, we also found that PDGFRB is one of the components of a complex that includes Beclin 1 and VPS34.

The results of these tissue-based analyses provide new insights into sunitinib's mechanism of action in SFT patients.

## INTRODUCTION

Solitary fibrous tumours (SFTs) are ubiquitous but rare soft tissue sarcomas bearing the *NAB2/STAT6* fusion gene that are characterised by a spectrum of “usual”, “malignant” and “dedifferentiated” variants [1–3]. Most SFTs fall into the so-called “usual” category and can be cured by means of complete surgical resection, but 10–15% behave aggressively and lead to local recurrences and/or distant metastases. Advanced SFTs are sensitive to sunitinib [4–8], although the rare dedifferentiated variant, which is currently regarded as a genetically reprogrammed, highly instable SFT [9], seems to be less sensitive to anti-angiogenic agents [4, 10].

Other drugs such as bevacizumab, sorafenib, pazopanib and IGF1R inhibitors [5, 6, 11–14] have also proved to be efficacious in treating advanced SFTs, but the complementary nature of the receptor tyrosine kinases (RTKs) activated in SFTs and the RTKs inhibited by sunitinib [5] suggests that sunitinib should be more effective. Moreover, stromal components such as the PDGFRB-expressing pericytes and the VEGFR2-expressing endothelial cells may be further targets [4]. Finally, it has recently been pointed out that the tumour immune contexture of SFTs changes in response to sunitinib, and that the host immune response contributes to the drug's efficacy [15]. However, the antitumoral efficacy of sunitinib is transient, and it can be hypothesised that the reduced blood flow and autophagy promoted by prolonged treatment act as adaptive mechanisms that ultimately lead to resistance [16].

It has been reported that a number of functionally different forms of autophagy are induced by anti-cancer drugs and radiation [17], and *in vitro* experiments have shown that sunitinib may induce either cytoprotective [18] or cytotoxic autophagy [19, 20]. Furthermore, the sequestration of sunitinib by lysosomes [21–23], or of Beclin 1 by RTKs [24, 25], causes defective/inhibited autophagy that may ultimately lead to dedifferentiation and the development of resistance by increasing genomic instability [26].

The aim of this study was to investigate the events that decrease the response to sunitinib and favour the development of sunitinib resistance in malignant SFTs by examining surgical samples taken from sunitinib-treated patients, a primary cell culture, and a stabilised cell line.

## RESULTS

### Sunitinib-induced changes in surgical samples of tumoral tissue

In order to make a thorough review of the changes induced by sunitinib, and their meaning in terms of response/resistance, we extensively examined three post-sunitinib surgical specimens obtained from two sunitinib-responsive patients with malignant SFT, and evaluated the characteristics of one tumour tissue specimen obtained from one of the patients before sunitinib treatment. No pre-sunitinib tissue was available from the other patient, but she provided tumour specimens taken after a first period of sunitinib treatment and after a sunitinib rechallenge.

**Figure 1:**
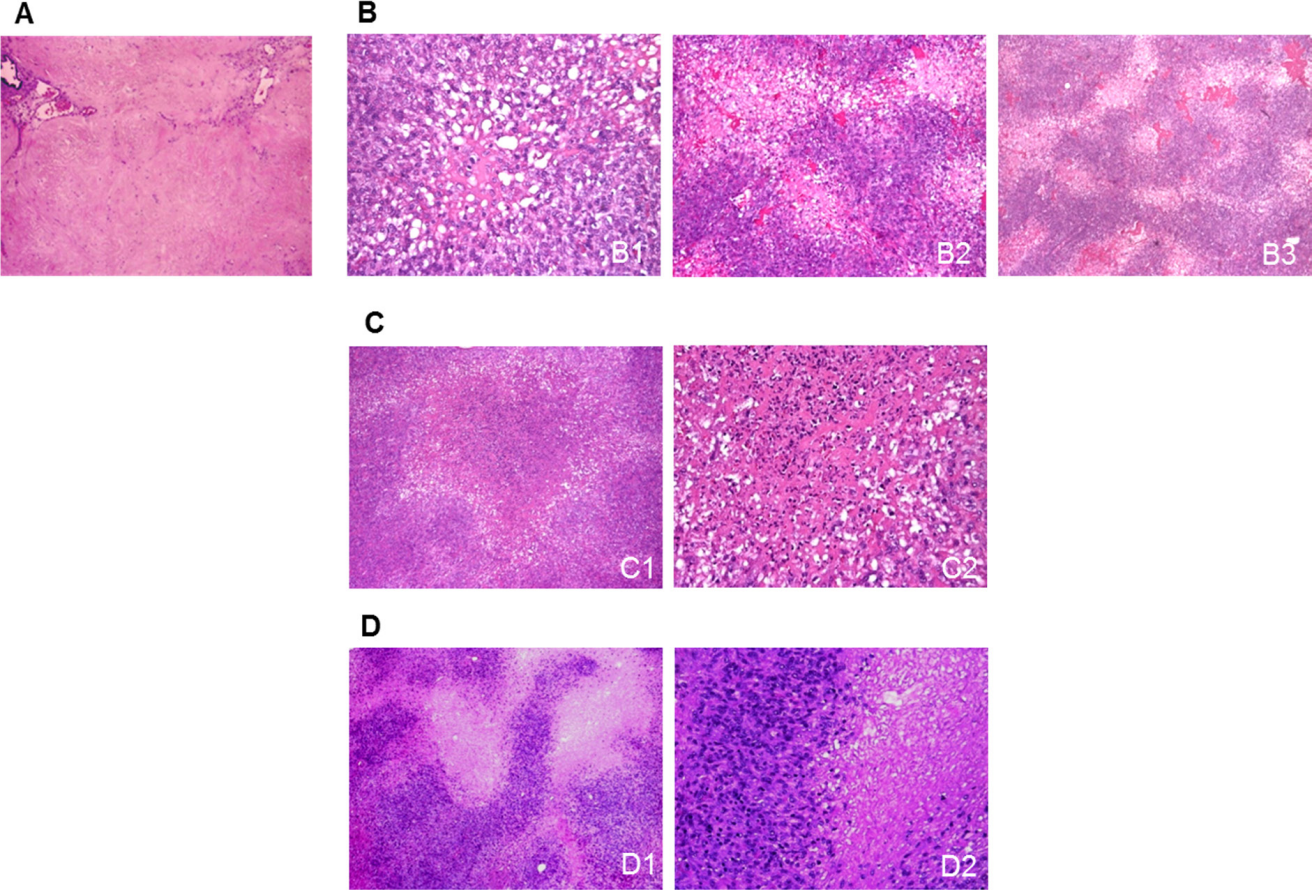
Sunitinib-induced morphological changes in surgical samples (**A**) A highly depleted cell area characterised by the presence of an eosinophilic proteinaceous matrix and scattered ectatic vessels. (**B**) Clear areas made up of cells showing cytoplasmic microvesicular swelling surrounded by more crowded cells arranged around a paucicellular (B1) or empty core (B2) that had a twisted appearance at low magnification (B3). (**C**) The empty cores were occasionally enlarged and filled by tumoral cells with picnotic nuclei, an inflammatory component (C1), and necrotic spots (C2). (**D**) The clear areas were sometimes replaced by discrete necrotic foci, as shown at low (D1) and higher magnification (D2).

The main post-sunitinib changes in both patients were rare, small (0.3–0.6 cm in diameter), randomly arranged and highly depleted cellular areas enriched in proteinaceous matrix (Figure 1A) and very frequent clear areas of sparsely distributed tumoral cells with cytoplasmic microvesicular alterations surrounded by more crowded cells. In some cases, the central areas were replaced by empty cores that had a twisted appearance at low magnification (Figure 1B). The empty cores were occasionally enlarged and filled by tumoral cells with picnotic nuclei, an inflammatory component and necrotic spots (Figure 1C) or, more frequently, entirely filled by necrosis (Figure 1D).

**Figure 2:**
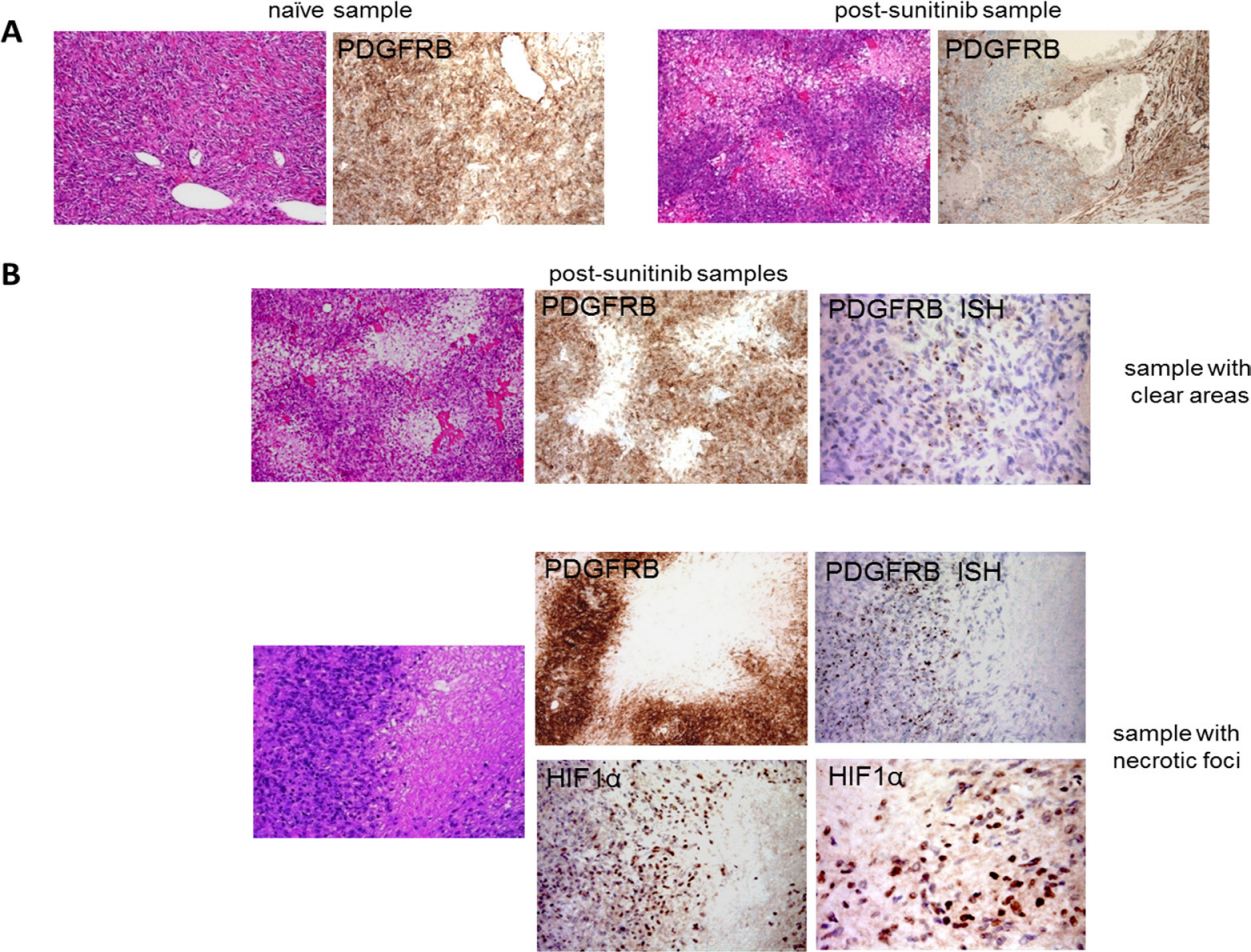
Sunitinib-induced changes in the morphology of tumour cells and their PDGFRB and HIF1α expression (**A**) H & E and IHC analyses of PDGFRB expression in a sunitinib-naïve and a post-sunitinib sample obtained from patient No. 1 showing clear areas and decreased PDGFRB expression in the latter. (**B**) IHC and ISH comparison of the clear areas and necrotic foci showed that the latter were characterised by increased PDGFRB expression and the appearance of HIF1α expression.

In order to investigate the effects of sunitinib on tumour cells and vessels, the clear areas and necrotic foci were compared with each other and with the sunitinib-naïve samples from patient No. 1. IHC showed decreased PDGFRB expression (Figure 2A) in all of the areas of the treated samples except those with necrotic foci. Remarkably, the tumoral cells surrounding the areas of necrosis expressed more PDGFRB protein as revealed by immunohistochemistry (IHC), and *PDGFRB* mRNA as revealed by *in situ* hybridisation (ISH) than those in the clear areas (Figure 2B). Furthermore, the cell boundary immediately encircling the necrotic areas showed HIF1α up-regulation (Figure 2B).

The expression of VEGFR2 (a marker of capillary endothelial cells) and nestin (a marker of pericytes) was significantly decreased in all of the post-sunitinib samples (Supplementary Figure 1), thus corroborating the activity of sunitinib on the tumour micro-environment.

**Figure 3:**
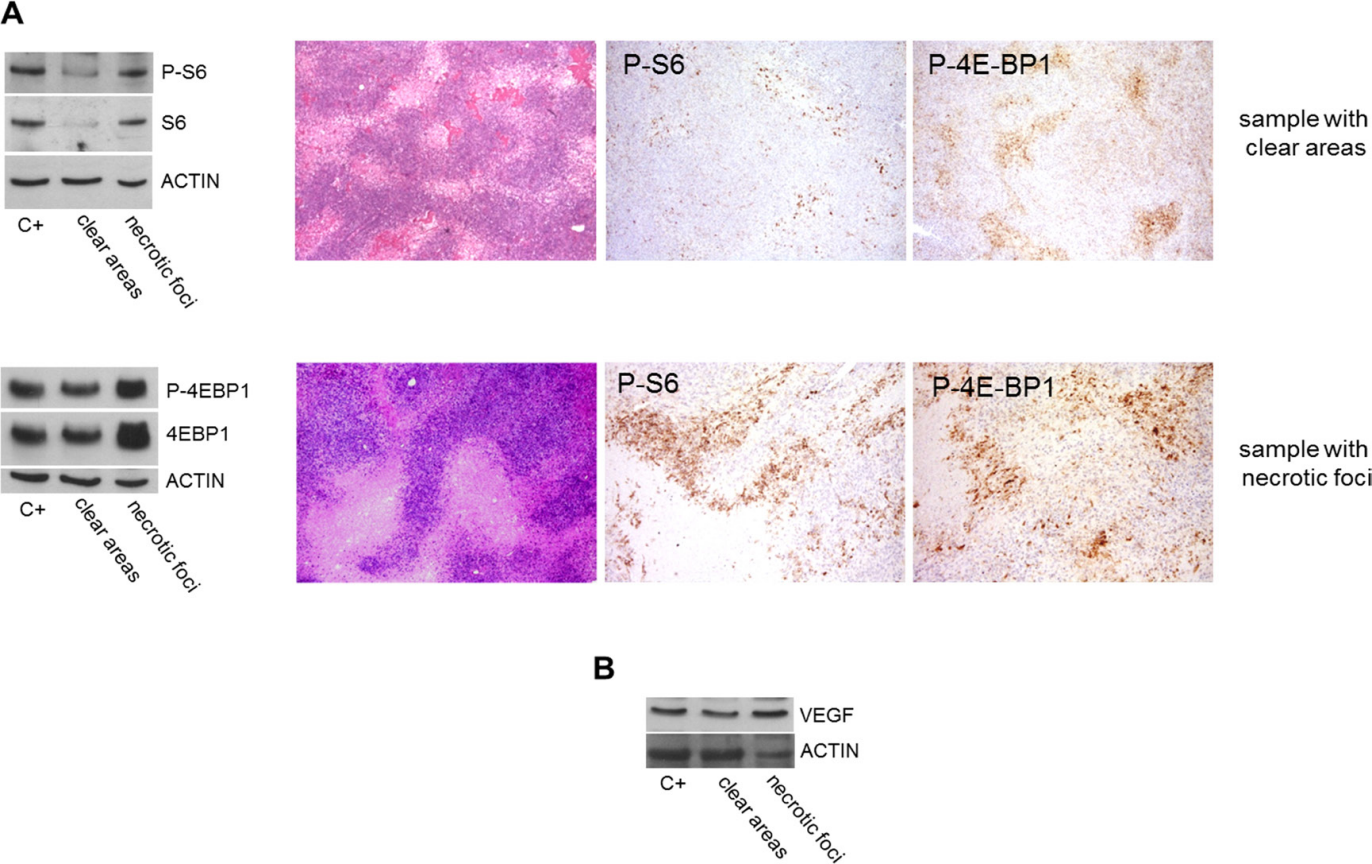
Comparison of mTOR effector phosphorylation and VEGF expression in post-sunitinib samples with clear areas or necrotic foci (**A**) WB revealed increased S6 and 4E-BP1 phosphorylation/expression in the sample with necrotic foci, and IHC confirmed the phosphorylation. (**B**) WB also showed that VEGF was more expressed in the sample with necrotic foci.

As there was insufficient cryopreserved material to verify the phosphorylation status of PDGFRB and VEGFR2 by means of immunoprecipitation or phospho-RTK arrays, we investigated the phosphorylation of the mTOR effectors S6 and 4E-BP1 as indirect markers of RTK activation. They were more phosphorylated in the post-sunitinib samples with necrotic foci than in those with clear areas (Figure 3A).

As PDGFRB and VEGFR2 are sustained by an autocrine/paracrine activation loop [4], and VEGFA can contribute to PDGFRB activation [27, 28], we investigated *PDGFB* and *VEGFA* expression by means of qRT-PCR. The level of *PDGFB* expression was the same in the post-sunitinib samples (regardless of whether they were characterised by clear areas or necrotic foci) but, in line with the presence of high HIF1α levels, the samples with necrotic foci showed increased VEGFA expression (data not shown), a finding that was confirmed by WB (Figure 3B).

Taken together, these findings suggest that the increased devascularisation induced by anti-angiogenic treatment may favour the development of severe hypoxia paralleling the appearance of necrotic foci, and the up-regulation of HIF1α, PDGFRB, mTOR effector activation and VEGFA expression. This is in line with the hypothesis that the clear areas correspond to a pathological response consistent with sunitinib efficacy, whereas the necrotic foci correspond to pathological progression.

### Sunitinib-induced autophagy and its role in response/resistance

**Figure 4:**
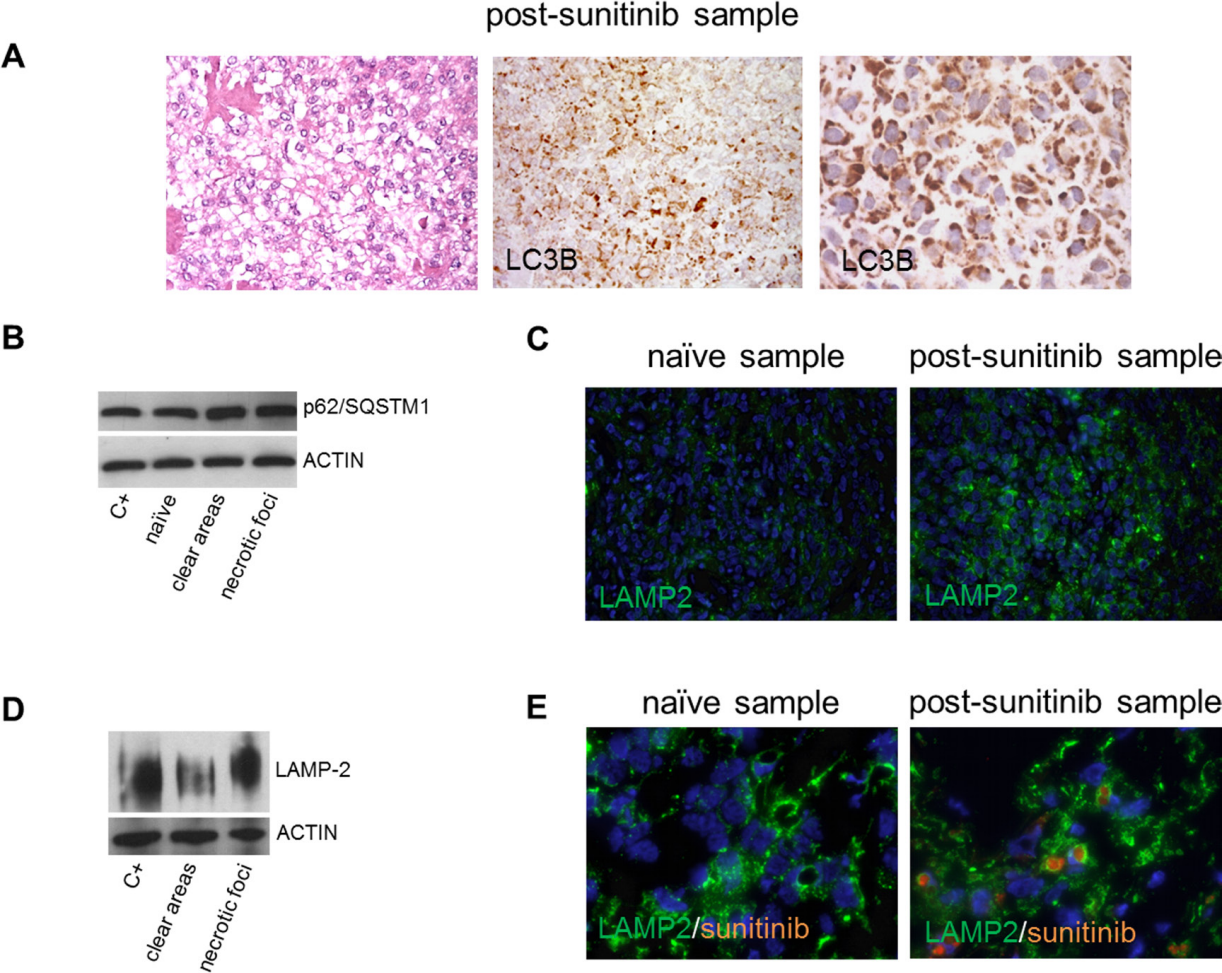
Sunitinib-induced autophagy in surgical specimens (**A**) LC3B-immunolabelled tumoral cells showing microvesicular cytoplasmic swelling (low and higher magnification) in a representative post-sunitinib sample with a clear/responsive area. (**B**) WB showing that p62/SQSTM1 was more expressed in post-sunitinib than in naïve samples. (**C**) IF of sunitinib-naïve and post-sunitinib samples showing the over-expression of LAMP2 and apparently larger lysosomes in the latter. (**D**) WB showing that LAMP2 was more expressed in a post-sunitinib sample with necrotic/progressive foci than in a post-sunitinib sample with clear/responsive areas. (**E**) IF of LAMP2 (green) and sunitinib (orange autofluorescence): the orange staining in the green labelled lysosomes indicates the localisation of sunitinib in the lysosomes in a post-sunitinib sample.

The presence of microvesicular swelling in the cytoplasm of tumoral cells in the clear/responsive areas suggests autophagosome induction (Figure 4A) and, as it is known that sunitinib induces autophagy [18–20], we assessed its occurrence and role in drug response.

### Surgical samples

IHC and Western blotting (WB) were respectively used to investigate the autophagy markers LC3B and p62/SQSTM1 in the surgical samples. IHC showed that the level of LC3B expression was low in the sunitinib-naїve samples (data not shown) in keeping with basal autophagy, whereas its expression was greater in all of the post-sunitinib samples regardless of the type of sunitinib-induced changes (clear areas or necrotic foci) (Figure 4A). WB showed that p62/SQSTM1 (an adaptor for delivering autophagy substrates to autophagosomes that is degraded in autolysosomes) was also more expressed in the post-sunitinib samples (Figure 4B).

These results confirmed the presence of autophagy, but did not allow us to distinguish whether it was complete or defective, although the accumulation of p62/SQSTM1 suggested the latter. The completion of autophagy requires functional lysosomes and, as sunitinib sequestration inhibits lysosomal function [21–23], we investigated the expression of LAMP2, which is a known marker of greater lysosomal volume in the case of dysfunction. Immunofluorescence (IF) analysis showed that all of the post-sunitinib samples had a larger lysosomal compartment and higher LAMP2 levels than the naїve samples (Figure 4C). It is also worth noting that WB-revealed LAMP2 expression in two post-sunitinib samples clearly indicated greater LAMP2 expression in the sample with necrotic/progressive foci (Figure 4D), and IF showed that LAMP2 co-localised with sunitinib in the post-sunitinib samples (Figure 4E).

Taken together, these results suggest that sunitinib induces defective autophagy mainly in necrotic/progressive foci.

### Stabilised cell line

Cells from the stabilised cell line were treated with sunitinib in order to establish the nature of the sunitinib-induced autophagic flux. It proved to be complete insofar as the cells showed a progressive increase in LC3B and decrease in p62/SQSTM1 levels in comparison with untreated cells (Supplementary Figure 2A), a finding that was supported by the increase in LC3B levels when the cells were simultaneously treated with sunitinib and chloroquine (a pharmacological inhibitor of autophagy) (Supplementary Figure 2B). Sunitinib treatment also led an increase in LAMP2 expression (Supplementary Figure 2C). Taken together, these findings suggest that, despite its interference with lysosomes, sunitinib does not block autophagy in this model.

In an attempt to distinguish whether this complete autophagy was cytotoxic and favoured a drug response, or cyprotective and induced drug resistance, we treated the cells with sunitinib or sunitinib combined with chloroquine for 24 hours. The presence of cleaved caspase-3 (Supplementary Figure 2D) showed that apoptosis was induced by a sunitinib dose of 10 μM and even more by a dose of 20 μM, a finding that is consistent with the tumoral cell apoptosis found in the post-sunitinib surgical samples (Supplementary Figure 3). Surprisingly, apoptosis was not modified by the addition of chloroquine and subsequent autophagy blockade (Supplementary Figure 2E), which suggests that the autophagy was neither cytotoxic nor cytoprotective, but simply non-protective as has been previously observed in other models [29]. These findings support the idea that sunitinib can induce both complete autophagy and autophagy-independent apoptosis in this model.

### Primary cell culture

As primary cell cultures usually resemble the tumour from which they originate more closely than virus-stabilised cell lines, we treated the primary cell culture with sunitinib 2.5 μM and found that it increased the levels of both LC3B and p62/SQSTM1, which accumulated in a time-dependent manner rather than being degraded (Supplementary Figure 4A). The trend was similar after 48 hours' treatment (Supplementary Figure 4B), which suggests the induction of defective autophagy. Furthermore, WB showed that LAMP2 expression was higher in the sunitinib-treated than in the untreated cells (Supplementary Figure 4C).

Finally, in order to investigate the effect of sunitinib on apoptosis, we evaluated the expression of cleaved caspase-3: exposure to sunitinib 2.5 μM did not lead to apoptotic cell death after 48 hours (Supplementary Figure 4D) or after up to five days' treatment with sunitinib 5 μM (data not shown). Unfortunately, we did not have enough cells to test higher sunitinib doses, and therefore cannot exclude the possibility that a dose of 10 or 20 μM may induce cell death.

Taken together, the primary cell culture data are in line with the hypothesis that defective autophagy is a possible cause of sunitinib resistance and tumour progression.

### Tumour-infiltrating immune cells in post-sunitinib samples

Given that: i) autophagy not only preserves cell homeostasis, but also shapes cell immunity and influences the differentiation and activation of myeloid and lymphoid cells [30]; ii) sunitinib has strong immunomodulating effects on the periphery and tumour sites of SFTs [15]; iii) our stabilised cell line readouts suggested the induction of non-protective autophagy, which has been found to elicit a local immune response *in vivo* [17]; and iv) the necrotic/progressive foci in our surgical specimens showed the increased VEGFA availability and the expression of HIF1α, both of which are known to mediate immunosuppressive function [31–33], we investigated the distribution of immune-related cells in post-sunitinib lesions by comparing the samples characterised by clear/responsive areas with those characterised by necrotic/progressive foci.

**Figure 5:**
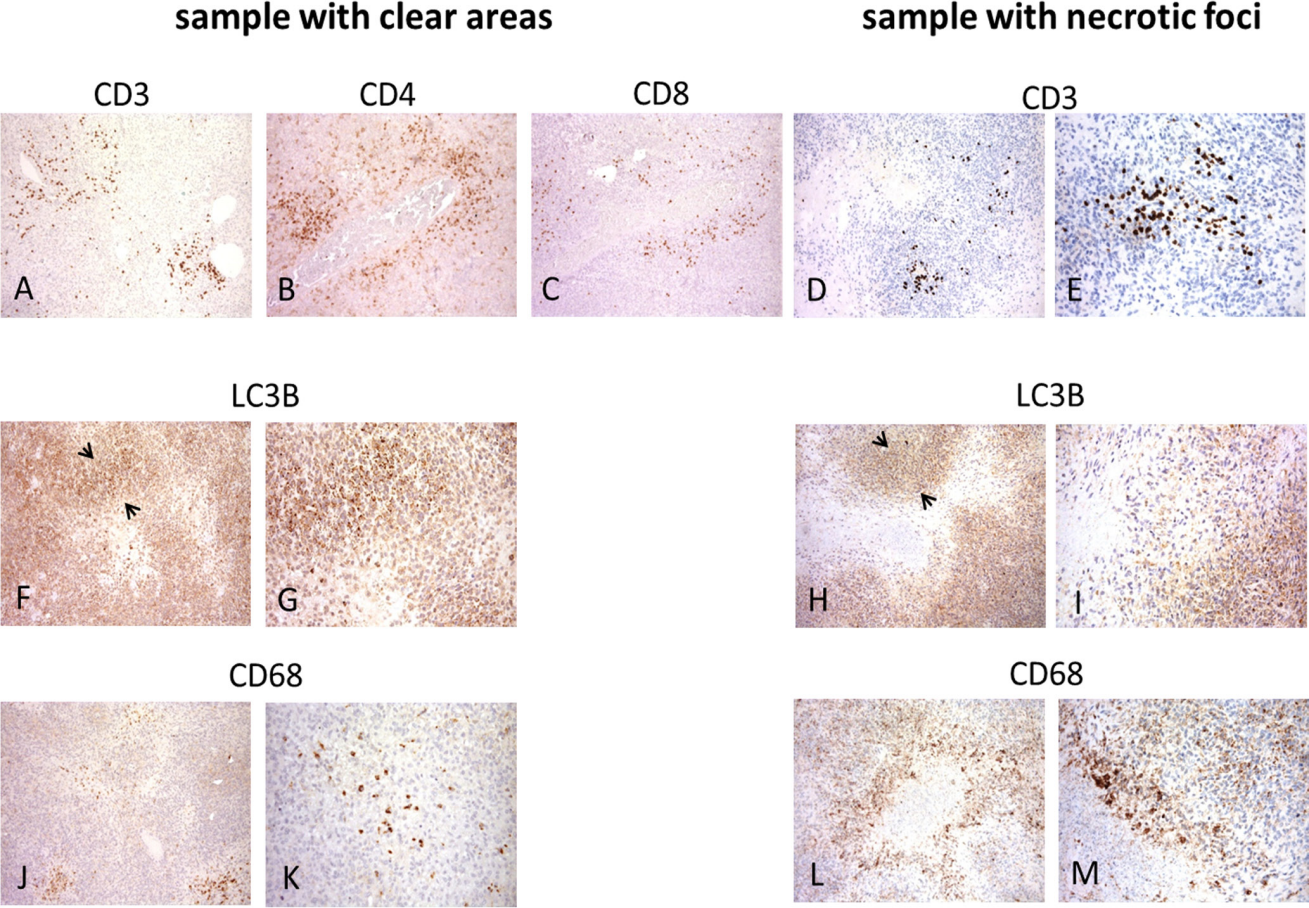
Distribution of immune T, myeloid and LC3B-immunolabelled cells in post-sunitinib samples Immunolabelled T cells were mainly located around the vessels in the sample with clear/responsive areas and the sample with necrotic/progressive foci (**A–E**), whereas LC3B mainly immunodecorated the outside boundary (see arrowheads) of tumour cells around paucicellular/empty cores (**F, G**) or necrotic foci (**H, I**). CD68-immunolabeled cells were restricted to the paucicellular/empty cores in the sample with clear/responsive areas (**J, K**), and mainly decorated the inside boundary of the cell layer surrounding the necrotic areas in the sample with necrotic/progressing foci (**L, M**).

In relation to lymphoid infiltration, there were no major differences in the distribution of CD3+ cells, which were mainly located around the vessels (Figure 5A–5E), and no selective enrichment in Foxp3+ regulatory T cells in the necrotic/progressive foci (data not shown), thus confirming our previous findings [15]. There were also no differences in LC3B staining, which mainly decorated the outside margins of the tumoral cells around the paucicellular/empty cores of the clear/responsive areas (Figure 5F, 5G) and around the necrotic/progressive foci (Figure 5H, 5I).

However, there were differences in CD68 staining: the CD68-immunolabelled cells in the clear/responsive areas were restricted to the paucicellular/empty cores and had the round morphology we have previously found to be associated with M1-like macrophages infiltrating sunitinib-treated tumours [15] (Figure 5J, 5K), whereas CD68 mainly decorated the inside margins of the cell layer surrounding the necrotic areas, and there were no CD68+ cells in the central core (Figure 5L, 5M).

**Figure 6:**
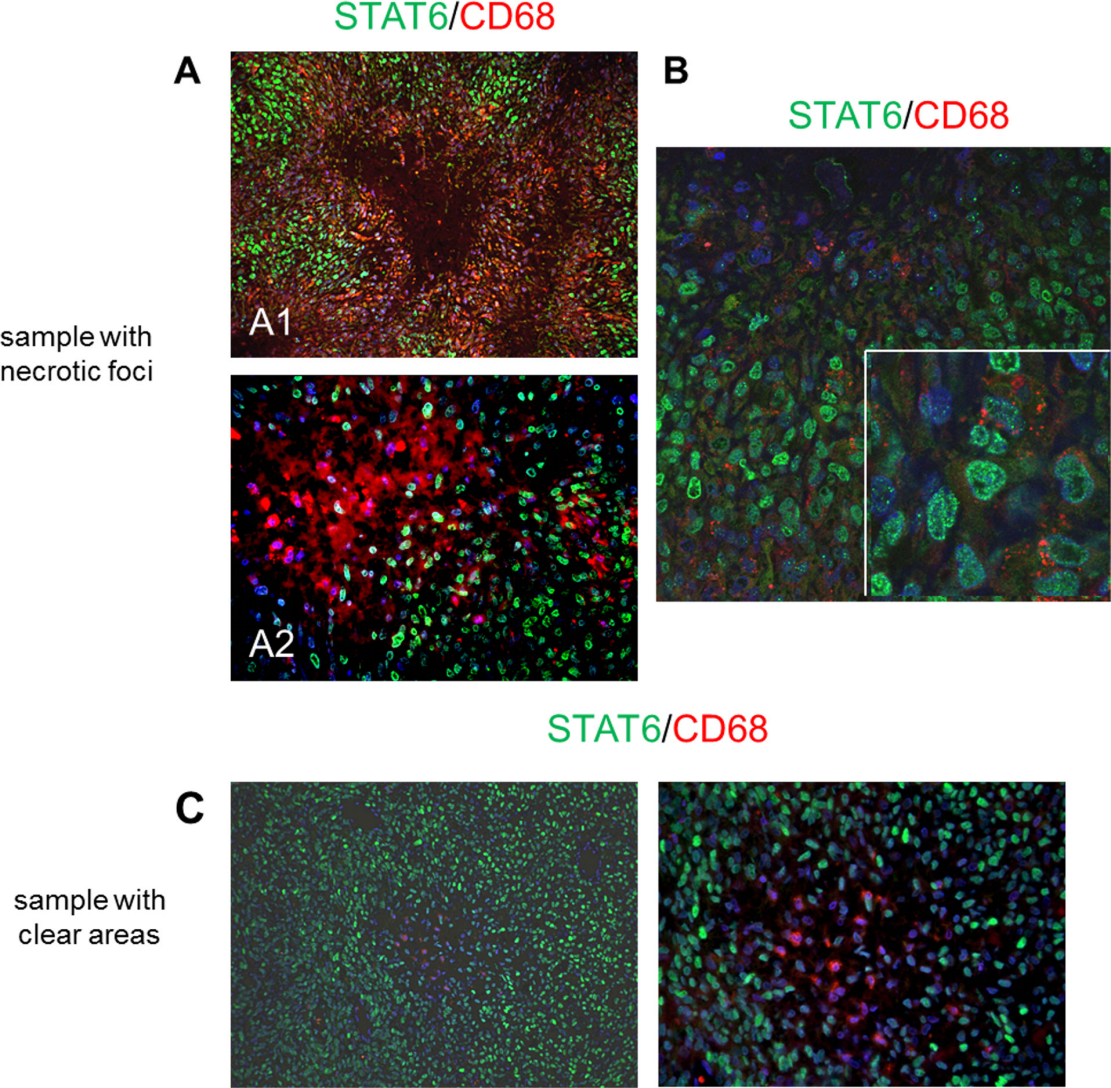
Distribution of STAT6- and CD68-positive cells in post-sunitinib samples revealed by means of IF and confocal microscopy (**A**) STAT6 (green) and CD68 (red) fluorescence immunolabelling in the sample with necrotic/progressive foci: note the nuclear presence of STAT6-positive tumour cells showing cytoplasmic CD68 immunodecoration in the merge. Low (A1) and higher magnification (A2). (**B**) Confocal microscopy confirmed the co-expression of nuclear STAT6 (green) and cytoplasmic CD68 (red): the insert shows a magnified image of the merge. (**C**) STAT6 (green) and CD68 (red) fluorescence immunolabelling in the sample with clear/responsive areas: none of the CD68-expressing cells inside the paucicellular core show STAT6 positivity.

This particular distribution led us to investigate the nature of these CD68+ cells further using IF and confocal microscopy, which showed that a number of cells with CD68-positive cytoplasm in the necrotic/progressive foci had nuclei immunolabelled with STAT6 antibody (Figure 6). Thus, the tumour cells had acquired this lysosomal/endosomal-associated membrane glycoprotein belonging to the family of scavenger receptors, whereas no double-positive cells were revealed by IF using an antibody specific for CD14, a marker further defining macrophages/myeloid cells (data not shown).

### Beclin 1 binding to PDGFRB

As it has been found that there is a dynamic Beclin 1-VPS34-protein binding network in which Beclin 1 acts as a central hub of autophagy regulation [34, 35] and that RTKs also inhibit autophagy insofar as phosphorylated EGFR and HER2 co-immunoprecipitate with Beclin 1 to suppress autophagy [24, 25], we investigated whether phosphorylated PDGFRB can interfere with autophagy by sequestering Beclin 1 and preventing it from binding to VPS34.

### Surgical sample

As expected, it was found that the PDGFRB immuno-precipitated from a sunitinib-naїve malignant SFT sample was phosphorylated, and that the immunoprecipitation of Beclin 1 led to its co-immunoprecipitation with PDGFRB and VPS34 (Supplementary Figure 5A). However, it was surprising that VPS34 was also co-immunoprecipitated when PDGFRB was immunoprecipitated, thus supporting the idea that the complex actually consisted of PDGFRB, Beclin 1 and VPS34 (Supplementary Figure 5A). We used the stabilised cell line in order to investigate this complex with and without sunitinib treatment.

### Stabilised cell line

The cells were left untreated or treated with sunitinib 10 μM for 24 hours which, as shown in Supplementary Figure 5B, is a sufficient dose and time to switch the receptor almost completely off. When Beclin 1 was immunoprecipitated, PDGFRB was co-immunoprecipitated regardless of its state of activation (Supplementary Figure 5C), and VPS34 was co-immunoprecipitated in untreated cells (in the presence of phosphorylated PDGFRB) and the treated cells (in which PDGFRB was switched off) (Supplementary Figure 5C). These findings suggest that SFT cells express the Beclin-1/PDGFRB/VPS34 complex, but further studies are required in order to establish whether the complex functions differently (possibly by means of a conformational regulation-based mechanism) in the presence of phosphorylated and non-phosphorylated PDGFRB.

## DISCUSSION

This hypothesis-generating study was based on a thorough and comprehensive analysis of four surgical specimens (one sunitinib-naïve and three post-treatment specimens) obtained from two patients with malignant SFTs who respectively showed signs of a RECIST partial response (PR) and stable disease (SD) after progression.

The findings indicate that sunitinib reduces the vascular supply network and inhibits tumoral cells in malignant SFTs. They also show that sunitinib-treated SFT cells are constantly evolving and that, although cell death eliminates a sizeable number of cancer cells in sunitinib-treated patients, hypoxia-inducing drug pressure accelerates cancer progression by promoting the repopulation of cancer cells with a more aggressive phenotype. Finally, they provide evidence that sunitinib induces autophagy associated with a local immune response, and that *in vitro* models only partially reflect what happens in SFT patients.

Analysis of the surgical samples showed that sunitinib induces two different signatures, one of which is morphologically and functionally consistent with a drug response, and the other with the development of resistance. The more frequently encountered picture is marked by clear/responsive areas and the attenuation/loss of PDGFRB expression, decreased mTOR signalling, and strong LC3B immunopositivity, all of which are consistent with the induction of autophagy and a drug response. The samples with necrotic/progressive foci showed HIF1α expression, increased PDGFRB and VEGFA levels, the phosphorylation of mTOR effectors, and the expression of LC3B, which can be attributed to defective autophagy and contributes to drug resistance.

In an attempt to gain insights into sunitinib-induced autophagy and its relationship with drug response or resistance, we performed *in vitro* experiments using a stabilised cell line and a primary cell culture. However, the results were not unequivocal because neither model paralleled *in vivo* tumour behaviour. Sunitinib induced a complete autophagic flux and apoptosis only in the stabilised cell line (which seemed to be a model of drug sensitivity), whereas autophagy was defective in the primary cell culture and sunitinib failed to induce cell death.

Combined sunitinib and chloroquine treatment did not alter sunitinib-induced apoptosis in the stabilised cell line, a finding that is in line with a form of non-protective autophagy similar to that observed in breast cancer cells that are neither sensitised to nor protected against radiation [29]. It is also worth noting that this type of autophagy elicits an immune response that is critical for drug effectiveness, as demonstrated by *in vivo* experiments in which autophagy-deficient tumours were engrafted in immunocompetent mice [36].

We have previously shown that sunitinib temporarily relieves the immunosuppression of sunitinib-naїve patients [15], and the present findings confirm that the drug's efficacy parallels the appearance of a signature consisting of activated tumour-infiltrating lymphocytes (TILs), including CD3+, CD4+ and CD8+ cells, and CD68+ myeloid cells [15]. The molecular events driving this immune profile are still unclear but they certainly include the drug-induced immunogenic cell death (ICD) that has been described in the case of antineoplastic chemotherapies and is often associated with different forms of autophagy [30].

Comparisons of the samples with clear/responsive areas and those with necrotic/progressive foci showed that the latter were associated with a more immunosuppressive environment characterised by the increased local availability of VEGFA and HIF1α, which impairs the maturation/functional status of dendritic cells (and myeloid cells in general) [31–33] and switches their activity toward immune suppression. The necrotic foci were also devoid of CD68+ cells having the morphological features of myeloid cells with scavenger activity, whereas some of the CD68 positivity in the areas surrounding the necrosis was due to the presence of tumour cells, as demonstrated by their STAT6-positive nuclei. CD68 has hitherto unknown functional significance in SFT cells, although it is worth noting that the acquisition of macrophage markers by tumoral cells in many other human cancers is often associated with a more malignant phenotype and generally correlates with a worse prognosis [37–40].

It is interesting to note that this changed phenotype in our system selectively occurs in the tumoral cells located in the necrotic/progressive areas of the tumours (which is probably where defective autophagy takes place), and may therefore represent one of the first steps in the process leading to the generation of a resistant, dedifferentiated form of SFT. In immunological terms, it can be speculated that CD68+ tumoral cells acquire scavenger activity and participate in clearing dying autophagic cells through a non-canonical autophagy pathway involving the selective engagement of LC3 (the so-called “LC3-associated phagocytosis” or LAP) [41, 42]. However, this process is inefficient because they are not professional antigen-presenting cells [43] and they ultimately compete with these for the efficient uptake and presentation of ‘danger signals’. Consequently, CD68+ tumour cells represent a ‘negative’ regulatory mechanism that blunts or limits immune responses in necrotic/progressive foci.

Finally, our studies of a sample from a sunitinib-naїve patient showed that PDGFRB is one of the components of a complex that also includes Beclin 1 and VPS34. Unfortunately, the functional study of the untreated and sunitinib-treated stabilised cell line did not reveal any changes in PDGFRB, Beclin 1 or VPS34 binding, although we cannot rule out the possibility of conformational or post-translational modifications that warrant further investigations.

In conclusion, our findings provide morphological and functional explanations for the clinical differences in sunitinib activity observed in SFT patients [4–8], and indicate that the drug's effectiveness or detrimental action is dictated by the prevalence of clear/responsive areas (a marker of disease response) or necrotic/progressive foci (a marker of disease progression). They also show that effective autophagy can contribute to inducing a sunitinib response by activating an adaptive immune response, whereas defective autophagy favours resistance by counteracting the immune response triggered by the treatment [44] and causing genomic instability, and this coincides with an advanced form of malignant SFTs that may be a prerequisite of the dedifferentiated SFT variant in our model [9].

## MATERIALS AND METHODS

### Ethics statement

Investigation has been conducted in accordance with the ethical standards and according to the Declaration of Helsinki and according to national and international guidelines and has been approved by the authors' institutional review board. In particular, the study was approved by the Institutional Ethics Committee. The patients whose biological samples were included in the study gave their signed consent to donate the tissues remaining after the diagnostic procedures had been completed.

### Surgical specimens

Formalin-fixed paraffin-embedded (FFPE) and cryopreserved material was obtained from two patients with advanced and progressive malignant SFTs, who underwent surgery after being treated with sunitinib. Pre- and post-sunitinib surgical specimens were obtained from patient No. 1; no pre-sunitinib tissue was available from patient No. 2, but she provided tumour specimens taken after a first period of sunitinib treatment and after a sunitinib rechallenge.

Patient No. 1 experienced the intra-abdominal relapse of a pelvic SFT and was surgically treated before receiving sunitinib (sunitinib-naïve specimen 1A, 6 samples). After experiencing a further (peritoneal) relapse, he was continuously treated with sunitinib 37.5 mg/day for 10 months, which led to evidence of tumor growth arrest after progression (RECIST stable disease, SD), and was followed by the surgical resection of residual disease (post-sunitinib specimen 1B, 5 samples).

Patient No. 2 was affected by a pelvic relapse of SFT characterised by multiple peritoneal lesions, and was continuously treated with sunitinib 37.5 mg/day for six months, which led to a RECIST partial response (PR), and followed by surgery (post-sunitinib specimen 2A, 3 samples). She subsequently remained disease-free for eight months before experiencing an abdominal relapse that was continuously treated with the same sunitinib dose for eight months. The treatment led to RECIST SD, and the residual tumour was excised (post-sunitinib specimen 2B, 7 samples) but the patient relapsed five months later (no more material was available).

In both cases, the post-sunitinib excisions were performed seven days after stopping the drug and, as RECIST PR and SD after progression are both interpreted as indicating drug effectiveness, responsive histological changes were expected in all the post-sunitinib surgical specimens.

Cryopreserved material available from a third patient with a malignant SFT who had never been treated with sunitinib was used to investigate the Beclin 1/PDGFRB complex.

The diagnoses were confirmed by means of immunohistochemistry (IHC: nuclear STAT6 positivity) [9] and real-time polymerase chain reaction (RT-PCR: NAB2-STAT6 rearrangement) [9].

### Primary SFT cell culture

A primary cell culture was obtained from fresh tissue taken from the sunitinib-naїve surgical specimen of patient No. 1 as previously described [45]. The nuclear expression of STAT6 in paraffin-embedded cells was verified by means of IHC [9], and the cells were treated with sunitinib (Cat. No. S1042; Selleck Chemical, Houston, TX, USA).

### Stabilised SFT cell line

The primary cell line was stabilised by means of the retroviral delivery of SV40 large T-antigen (pWZL-neo Large T-Ag) in accordance with standard procedures [46]: a SV40 LgT-SFT bulk cell population was used throughout the study. The nuclear expression of STAT6 in paraffin-embedded cells was verified by means of IHC [9], and the cells were treated with sunitinib and chloroquine diphosphate salt (C6628; Sigma-Aldrich, St. Louis, MO, USA).

### FFPE material

#### Immunohistochemistry (IHC) and immunofluorescence (IF)/confocal microscopy

The conditions and primary antibodies are described in the Supplementary Data.

#### Bright field *in situ* hybridisation (ISH)

*PDGFRB* mRNA-based ISH was performed manually as described in the Supplementary Data.

### Cryopreserved material

#### Immunoprecipitation (IP) and Western blotting (WB)

Total proteins were extracted from the surgical specimens or cells as previously described [48]. The conditions, the primary and secondary antibodies, and the positive controls are described in the Supplementary Data.

#### Real-time PCR (qRT-PCR)

*PDGFB* and *VEGFA* were detected as previously described [50].

## Supplementary Materials


