# Supplementary material for: Sunitinib-induced morpho-functional changes and drug effectiveness in malignant solitary fibrous tumours
Source: Oncotarget. 2016 Feb 20;7(29):45015–26. doi: 10.18632/oncotarget.7523 (PMC5216702; doi:10.18632/oncotarget.7523)
Supplement: Supplementary file 1 [file oncotarget-07-45015-s001.pdf]

Sunitinib-induced Morpho-functional changes and drug effectiveness in malignant solitary fibrous tumours

Supplementary Materials

METHODS

FFPE material

Immunohistochemistry (IHC) and immunofluorescence(IF)/confocal microscopy

Primary antibodies and methodological conditions:

PDGFRB (#4564, 1:25), VEGFR2 (#2479, 1:300), phosphoS6$_{ser240-244}$ (#5364, 1:25), phospho-4E-BP1$_{thr37-46}$ (#2855, 1:25) (all purchased from Cell Signaling Technology, Danvers, MA), nestin (AM12086PU-N, 1:100, Acris Antibodies, Herford, Germany) and HIF1α (MAB5382, 1:50, Merck Millipore, Billerica, MA) were processed using the UltraviewDAB Detection Kit on the Ventana BenchMark ULTRA platform (Ventana Medical Systems, Tucson, AZ) in accordance with the manufacturer's instructions. LC3B (0231-100/LC3-SF10, 1:50, Nanotools, Teningen, Germany) was unmasked using citrate buffer, pH 6, for 30 minutes at 95°C, whereas cleaved caspase-3 (#9664, 1:300, Cell Signaling Technology) immunodecoration was carried out using a pressure cooker (antigen retrieval citrate buffer, SP1, 110°C, 15 minutes). The antibodies and conditions used for CD3, CD4, CD8 and CD68 immunodetection have been previously described [15].

The first part of the LAMP2 (sc-18822, 1:200, Santa Cruz Biotechnology, Santa Cruz, CA) IF protocol was performed using the Ventana BenchMark ULTRA platform, whereas the primary antibody (overnight at +4°C) and subsequent fluorescence secondary antibody (Alexa Fluor 488, Invitrogen, Waltham, MA) were manually incubated. The anti-cleaved caspase-3 IF protocol used the same antigen retrieval conditions as those used for IHC, followed by Alexa Fluor 488 secondary antibody incubation.

The co-localisation immunofluorescence analyses of CD14 (MS-1080-S1, 1:10, Thermo Scientific, Waltham, MA)/STAT6 (sc-621, 1:400, Santa Cruz Biotechnology) and CD68 (M0814, 1:3000, Dako, Glostrup, Denmark)/STAT6 were carried out using a pressure cooker (antigen retrieval EDTA buffer, SP1, 110°C, 20 minutes) followed by incubation with fluorescent secondary antibodies (Alexa Fluor 488 and Alexa Fluor 546). The samples were observed through a Leica DM6000B microscope equipped with a 100 W mercury lamp; excitation was obtained using Spectrum Green, Spectrum Orange and DAPI excitation filters. The images were acquired using 20x and 40x oil immersion objectives, and analysed using Cytovision software. The images from each channel were collected sequentially in order to limit fluorescence cross-talk. The published images show extended depth-of-field frames in stack with focal regions selected on the basis of their maximum intensity.

Confocal microscopy

The slides were examined by means of confocal microscopy (Leica TCS SP8 confocal microscope). The images were obtained using a 40x oil immersion lens and analysed using Image-Pro Plus v.7.0.1 software (MediaCybernetics).

Bright field *in situ* hybridisation (ISH)

PDGFRB mRNA-based ISH was performed using an RNAscope probe (#310020; Advanced Cell Diagnostics, Inc., Hayward, CA) and the RNAscope 2.0 detection kit – brown (#310033; Advanced Cell Diagnostics) as previously described [47]. The control probes for the bacterial gene 4-hydroxy-tetrahydrodipicolinate reductase (DapB) negative control and the housekeeping gene ubiquitin C (UbC) positive control for evidence of preserved RNA were also included in each case.

Cryopreserved material

Immunoprecipitation (IP) and Western blotting (WB)

The co-immunoprecipitation experiments were performed using incubation with anti-Beclin 1 antibody (sc-11427; Santa Cruz Biotechnology) as previously described [49], or anti-PDGFRB antibody (20 µl; sc-432; Santa Cruz Biotechnology). The proteins were separated by means of SDS-polyacrylamide gel electrophoresis, and transferred onto a PVDF membrane (IPVH304F0; Merck Millipore). The bands were detected using ECL Western blotting detection reagents and ECL hyperfilm (Amersham Biosciences, Milan, Italy).

Primary antibodies

PDGFRB (#4564, 1:1000), Phospho-S6 (#2215, 1:2000), S6 (#2217, 1:1000), phospho-4E-BP1 (#9455, 1:1000), 4E-BP1 (#9644, 1:1000), LC3B (#2275, 1:1000), p62/SQSTM1 (#5114, 1:1000), and Caspase-3 (#9662,

1:1000) (all purchased from Cell Signaling Technology); LAMP2 (sc-18822, 1:500) and Beclin 1 (sc-11427, 1:200) (purchased from Santa Cruz Biotechnology); phosphotyrosine (p-Tyr: 05-321, 1:3000) (Merck Millipore), VPS34 (38-2100, 1:250) (Zymed Laboratories Inc., South San Francisco, CA), and VEGF (RB-9031-P0, 1:1000) (Thermo Scientific). Actin antibody diluted 1:2500 (A2066; Sigma-Aldrich) was used to normalise the results.

The membranes were incubated with secondary anti-rabbit (sc-2004), anti-mouse antibodies (sc-2005) from Santa Cruz Biotechnology, and protein A, peroxidase-linked (NA9120; Amersham Biosciences) at the recommended dilutions.

The positive controls were: HeLa Whole Cell Lysate (sc-2200, Santa Cruz Biotechnology) for S6, 4E-BP1, LC3B, p62/SQSTM1, Beclin 1 and VPS34 detection; Jurkat Cell Extract Untreated (#9663S; Cell Signaling Technology) for LAMP2 and caspase 3; Jurkat Cell Extract Cytochrome C Treated (#9663S; Cell Signaling Technology) for cleaved caspase-3; the 2N5A cell line (derived from the NIH3T3 cell line expressing the *COL1A1-PDGFB* fusion that characterises dermatofibrosarcoma protuberans, which was kindly provided by Dr. Greco, Fondazione IRCCS Istituto Nazionale dei Tumori, Milan, Italy) for PDGFRB IP, and a protein extract from a surgical specimen of angiosarcoma for VEGF.

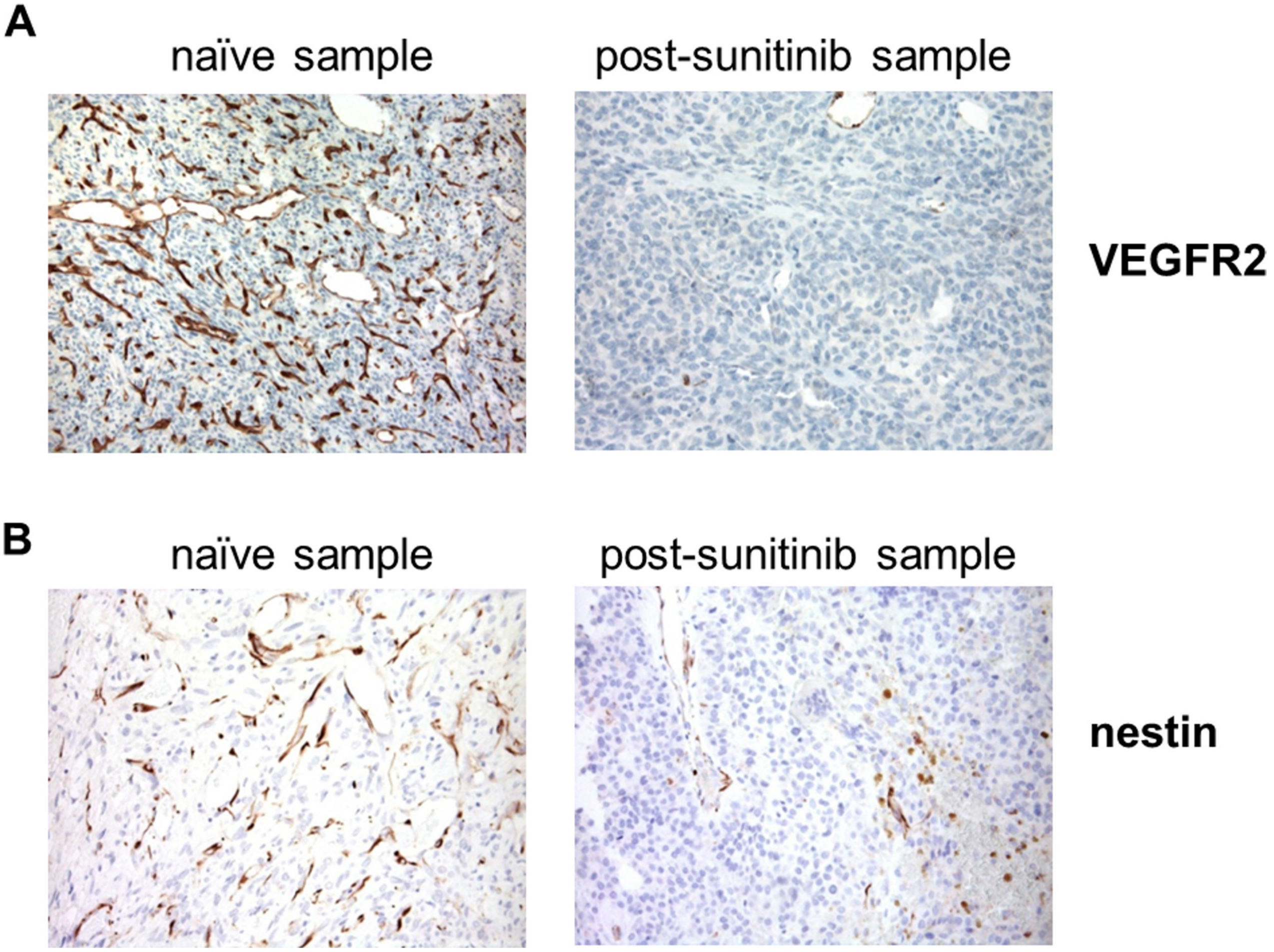

Supplementary Figure S1: Sunitinib-induced changes in vessels. (**A**) IHC analysis of VEGFR2 in sunitinib-naïve and post-sunitinib samples showing that treatment decreased the number of VEGFR2-positive endothelial cells. (**B**) IHC analysis of nestin in sunitinib-naïve and post-sunitinib samples showing that treatment decreased the number of pericytes immunolabelled with anti-nestin antibody.

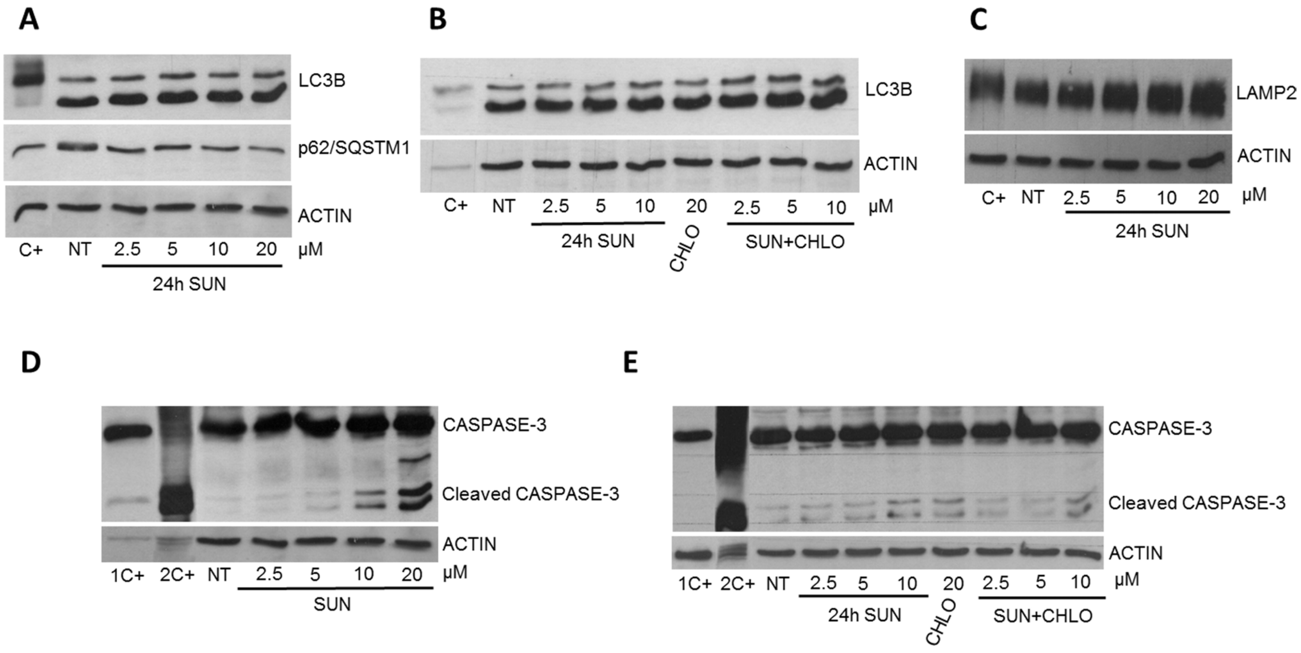

Supplementary Figure S2: Sunitinib-induced autophagy in the stabilised cell line. (**A**) WB analyses of autophagy markers in untreated (NT) and sunitinib-treated cells (SUN). Sunitinib treatment for 24 hours induced a dose-dependent decrease in the expression of p62/SQSTM1, and an increase in the expression of LC3B. (B) WB analysis of LC3B expression in untreated cells (NT), and cells treated with sunitinib (SUN), chloroquine (CHLO) or sunitinib + chloroquine (SUN+CHLO) for 24 hours. The greater expression of LC3B in the cells treated with the drug combination indicates that sunitinib induces a complete autophagic flux. (**C**) WB analysis of LAMP2 in untreated cells (NT) and cells treated with sunitinib (SUN) for 24 hours. Sunitinib induced a dose-dependent increase in LAMP2 expression. (**D**) WB analysis of cleaved caspase-3 expression in untreated cells (NT) and cells treated with sunitinib (SUN) for 24 hours in order to assess sunitinib-induced apoptosis: caspase-3 was cleaved at sunitinib doses of 10 and 20 µM. The positive controls for cleaved caspase-3 and caspase-3 were respectively Jurkat Cell Extract Cytochrome C treated (2C+) and untreated (1C+). (**E**) WB analysis of cleaved caspase-3 expression in untreated cells (NT) and cells treated with sunitinib (SUN), chloroquine (CHLO) or sunitinib + chloroquine (SUN+CHLO) for 24 hours in order to investigate the correlation between sunitinib-induced apoptosis and autophagy. The addition of chloroquine did not modify the level of cleaved caspase-3 expression. The positive controls for cleaved caspase-3 and caspase-3 were respectively Jurkat Cell Extract Cytochrome C treated (2C+) and untreated (1C+).

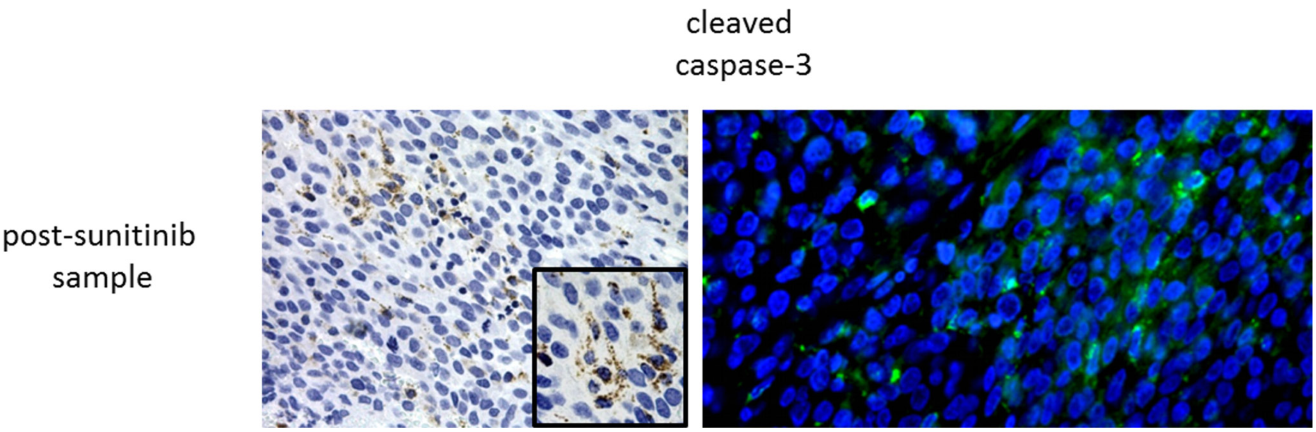

Supplementary Figure S3: Tumour cell apoptosis in surgical specimens. IHC (left) and IF (right) analyses of a representative post-sunitinib sample showing tumour cells positive for cleaved caspase-3.

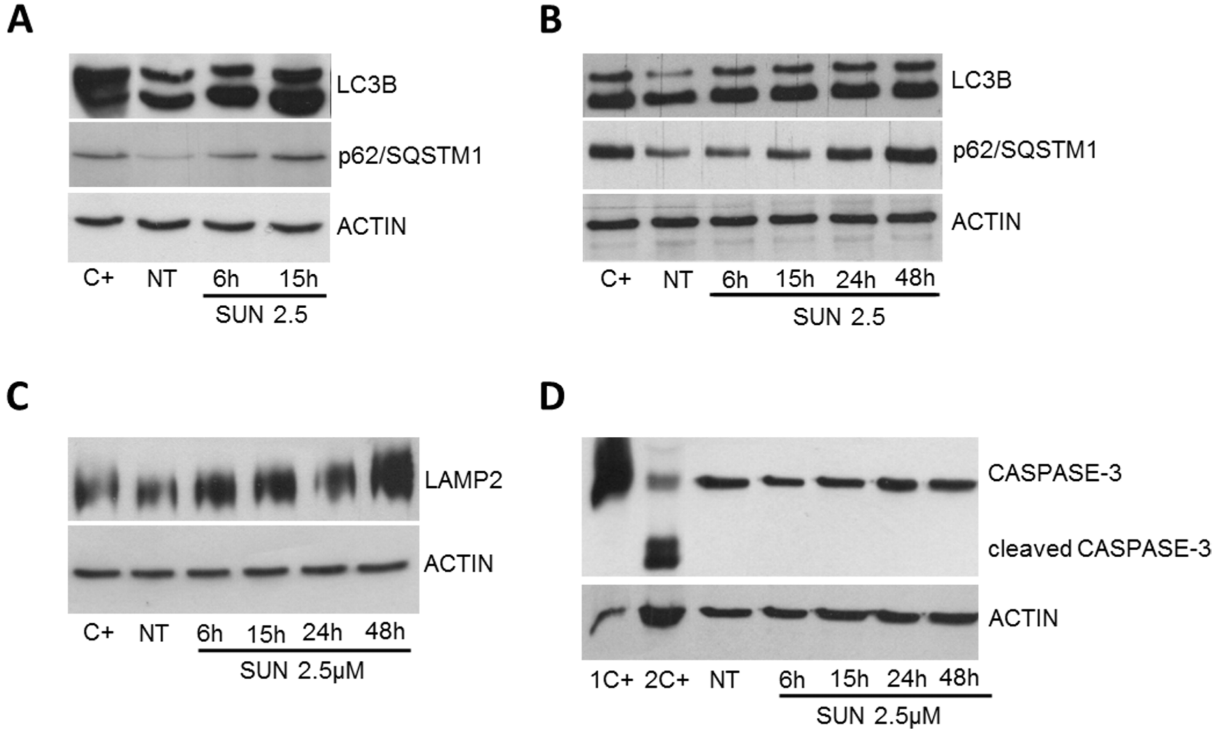

Supplementary Figure S4: Sunitinib-induced autophagy in primary cell culture. (**A**) WB analyses of autophagy markers in untreated cells (NT) and cells treated with sunitinib for up to 15 hours. Sunitinib 2.5 μM started to induce a time-dependent increase in LC3B and p62/SQSTM1 expression after six hours. (**B**) WB analyses of autophagy markers in untreated cells (NT) and cells treated with sunitinib for up to 48 hours. Sunitinib 2.5 μM induced a time-dependent increase in the expression of both LC3B and p62/SQSTM1. (**C**) WB analysis of LAMP2 in untreated (NT) and sunitinib-treated cells. Sunitinib 2.5 μM induced an increase in LAMP2 expression. (**D**) WB analysis of cleaved caspase-3 in untreated (NT) and sunitinib-treated cells. Sunitinib 2.5 μM did not induce caspase-3 cleavage. The positive controls for cleaved caspase-3 and caspase-3 were respectively Jurkat Cell Extract Cytochrome C treated (2C+) and untreated (1C+).

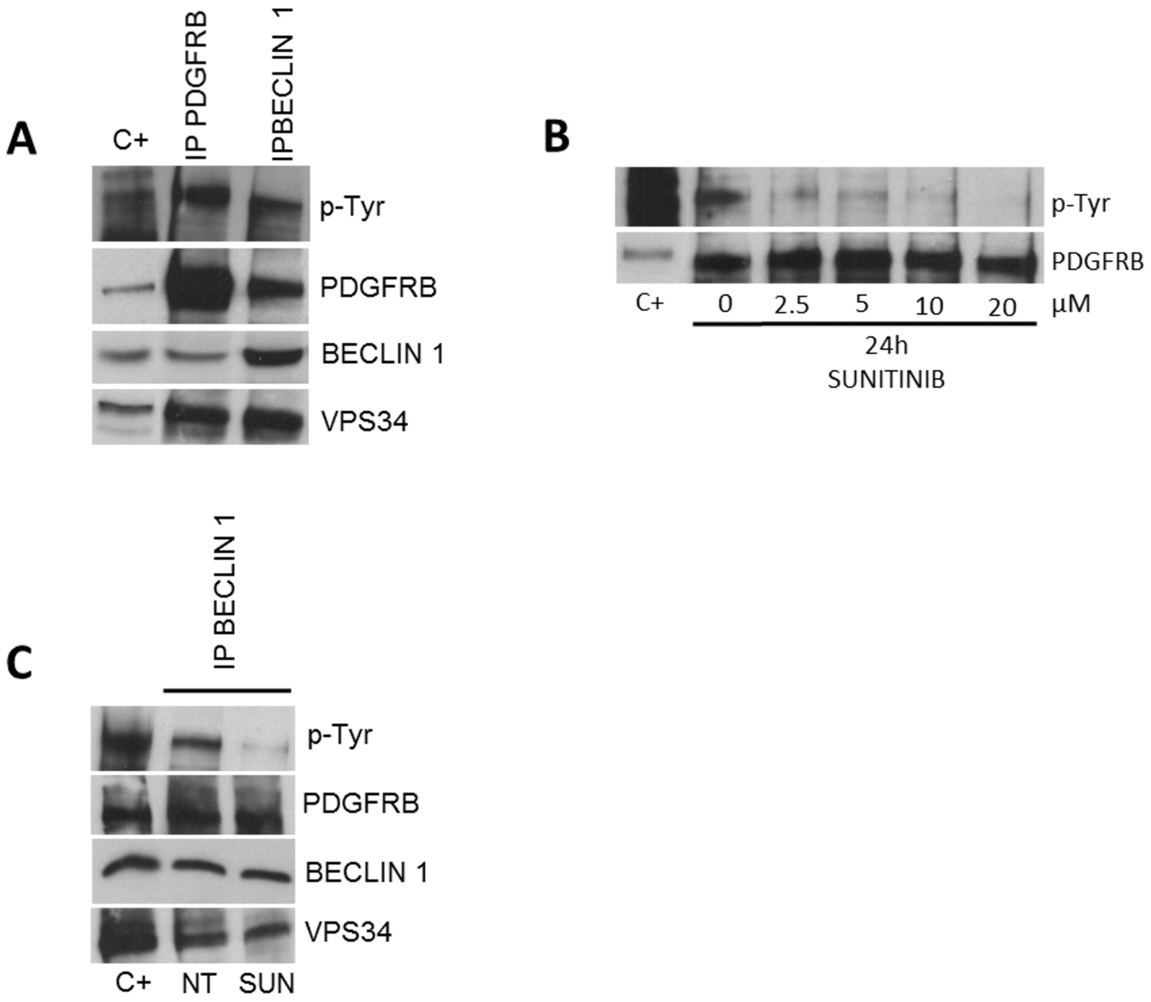

Supplementary Figure S5: Beclin 1 binding to PDGFRB. (**A**) IP of PDGFRB (lane IP PDGFRB) and Beclin 1 (lane IP Beclin 1) in a treatment-naïve surgical sample in order to investigate the co-immunoprecipitation of PDGFRB, Beclin 1 and VPS34. WB using antibodies against pTyr (to detect the phosphorylation status of PDGFRB), PDGFRB, Beclin 1 and VPS34 showing the presence of a complex of phosphorylated PDGFRB, Beclin 1 and VPS34. (**B**) IP/WB analysis of PDGFRB phosphorylation/expression in untreated cells (NT) and the stabilised sunitinib-treated cell line (SUN). After treatment, PDGFRB phosphorylation detected using p-Tyr antibody decreased in a dose-dependent manner, whereas its expression, detected using PDGFRB antibody remained unchanged. (**C**) IP of Beclin 1 in untreated cells (NT) and the stabilised cell line treated with sunitinib 10 µM for 24 hours (SUN) in order to investigate whether its co-immunoprecipitation with PDGFRB and VPS34 changes depending on whether PDGFRB is phosphorylated or not. WB using antibodies against p-Tyr, PDGFRB, Beclin 1 and VPS34 demonstrated that PDGFRB, Beclin 1 and VPS34 form a complex regardless of the activation status of PDGFRB.
